# P-1450. Cellular Correlates of Immunogenicity Following Standard- Versus High-Dose Influenza Vaccination in Adult Hematopoietic Cell Transplant Recipients Over Two Seasons

**DOI:** 10.1093/ofid/ofaf695.1636

**Published:** 2026-01-11

**Authors:** Haya Hayek, Justin Z Amarin, Joshua Simmons, Michael Ison, Steven A Pergam, James Chappell, Andrew J Spieker, Natasha B Halasa, Spyros A Kalams

**Affiliations:** Vanderbilt University Medical Center, Nashville, TN; Vanderbilt University Medical Center, Nashville, TN; Vanderbilt University Medical Center, Nashville, TN; Respiratory Diseases Branch, DMID/NIAID/NIH, Derwood, MD; Fred Hutchinson Cancer Center, Seattle, Washington; Vanderbilt University Medical Center, Nashville, TN; Vanderbilt University Medical Center, Nashville, TN; Vanderbilt University Medical Center, Nashville, TN; Vanderbilt University Medical Center, Nashville, TN

## Abstract

**Background:**

Adult hematopoietic cell transplant (HCT) recipients are at higher risk for severe influenza but often exhibit poor vaccine-induced antibody responses. While lymphocyte counts are a marker of immunogenicity, the role of specific B and T cell subsets remains unclear. We evaluated associations between baseline immune cell subpopulation counts and antibody responses to standard- versus high-dose influenza vaccination over two consecutive seasons.

**Figure:**
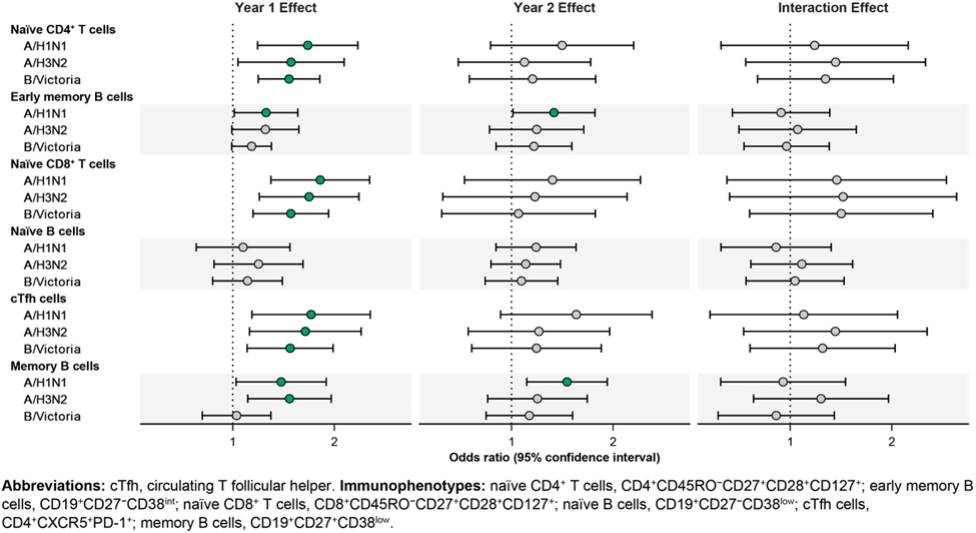
Associations between baseline immune cell counts and subsequent hemagglutination inhibition (HAI) antibody response in adult hematopoietic cell transplant recipients vaccinated over 2 consecutive years.

The forest plot displays point estimates (points) and confidence intervals (horizontal lines) from a model fit using generalized estimating equations for the association between baseline cell counts (log2 scale) and subsequent geometric mean HAI antibody titers measured 28–42 days after two doses of influenza vaccine. Estimates represent fold-change in geometric mean antibody titer between subgroups differing in baseline cell count by a factor of 2, adjusted for initial baseline antibody titer and vaccine dose. Results are shown separately for Year 1, Year 2, and the difference between years (with Year 2 as the reference), grouped by cell subpopulation and influenza immunogen (A/H1N1, A/H3N2, and B/Victoria). The dashed vertical line indicates the null value (fold-change of 1). Points in green indicate sufficient evidence of an association based on a nominal threshold of α=0.05.

**Methods:**

This secondary analysis included adults (≥18 years) 3–23 months post-allogeneic HCT who participated in a multicenter, double-blind, randomized controlled trial over two consecutive influenza seasons. Participants were randomized 1:1 to receive two doses of high-dose trivalent influenza vaccine or standard-dose quadrivalent influenza vaccine. Participants could re-enroll the following season to receive the same assigned vaccine. Mass cytometry was used to quantify 36 immune cell subpopulations from peripheral blood mononuclear cells collected before initial vaccination each year. Immunogenicity was assessed by serum hemagglutination inhibition (HAI) titers measured at baseline and 28–42 days after second vaccination. We modeled associations between select baseline immune cell counts (identified *a priori*) and subsequent HAI titers, including the interaction between baseline count and year, adjusting for initial baseline titer and vaccine dose.

**Results:**

Among 41 participants with paired data across both seasons, we identified higher baseline counts of naïve CD4^+^ and CD8^+^ T cells, early memory B cells, naïve T cells, circulating T follicular helper cells, and memory B cells to be significantly associated with higher post-vaccination HAI titers in Year 1. In Year 2, we found similar evidence for early memory and memory B cells. We did not identify statistically significant differences between these associations from Year 1 to Year 2 (Figure).

**Conclusion:**

Baseline counts of specific immune subpopulations at time of vaccination are associated with influenza vaccine-induced antibody responses in adult HCT recipients, particularly in the early post-HCT period. Studies to assess evolving post-HCT immune reconstitution effects on vaccine responsiveness are needed.

**Disclosures:**

Michael Ison, MD MS, UpToDate: Honoraria|Wiley: Honoraria Steven A. Pergam, MD, MPH, F2G: Participate in company sponsored clinical trial|Mundipharma: Participate in company sponsored clinical trial|Symbio: Participate in company sponsored clinical trial James Chappell, MD, PhD, Merck: Grant support for etiologic studies of acute respiratory illness in hospitalized children, Amman, Jordan Natasha B. Halasa, MD, CSL-Seqirus: Advisor/Consultant|Merck: Grant/Research Support

